# Supplementary material for: Impact of Climate Variability and Interventions on Malaria Incidence and Forecasting in Burkina Faso
Source: Int J Environ Res Public Health. 2024 Nov 8;21(11):1487. doi: 10.3390/ijerph21111487 (PMC11593955; doi:10.3390/ijerph21111487)
Supplement: Supplementary file 1 [file ijerph-21-01487-s001.zip › Supplementary material S1.pdf]

Model formulations

A-GARMA negative binomial

Generalized autoregressive moving average GARMA (p, q) negative binomial models were fitted to the malaria incidence data with orders p and q representing the autoregressive (AR) and moving average (MA) terms, respectively.

The number of malaria cases from all health facilities within a climatic zone in month *t=1… 84* during 2015-2021 follows a negative binomial distribution, $Y_t \sim NB(p_t, r)$ where $p_t = r/ r+\mu_t$; r is the dispersion parameter. We modeled the average number of malaria cases μ_t in t using a log link function, that is, $\log(\mu_t) = \log(N_t) + x_t'\beta + Z_t$, where $Z_t = \sum_{j=1}^{p} \Phi_j (g(y_{t-j}^{*}) - x_{t-j}') + \sum_{j=1}^{q} \theta_i (g(y_{t-j}^{*}) - \mu_{t-j})$, $x_t'\beta = \beta_0 + \beta_1 Rain_t + \beta_2 LST_t + \beta_3 ACT_t + \beta_4 Bednet_t$, $y_{t-j}^{*} = \max(y_{t-j}, c), 0 < c < 1$. $Rain_t$ and LST_t are the predictors for selected lags. N_t is the population size, β_k, $k=0,1,2,3,4$ are the regression coefficients quantifying the effect of the predictors on the current malaria cases, Y_t. $\Phi_j, j = 1, \dots p$ and $\theta_j, j = 1, \dots q$ are the auto-regressive and the moving average parameters, respectively.

We formulated the models within the Bayesian framework. Normal prior distributions were adopted for Φ_j and θ_i, that is $\Phi_j \sim N(0, \sigma_j^2)$, $\theta_j \sim N(0, \sigma_j^2)$, Gamma distribution with mean 1 and variance 100 was used for the parameter r, inverse-gamma priors for the variance parameter $\sigma_j^2 \sim IG(0.01, 0.01)$ and non-informative Gaussian distributions with mean 0 and variance 100 for the regression coefficients, i.e. $\beta_k \sim N(0, 10^2)$. We developed a separate model for each climatic zone.

B- Bayesian variable selection

Bayesian variable selection through stochastic search [36] was employed to identify the most appropriate lag for each climatic predictor, X_p, p=1,2 (i.e., Rainfall and LST). In particular, for each X_p, a categorical variable I_p, was introduced into the model and assigned values 0 for exclusion of the predictor from the model ($I_p = 1$), and inclusion as follows: lag 0 ($I_p = 2$), lag 1 ($I_p = 3$), lag 2 ($I_p = 4$),

lag 3 (I_p = 5), lag 01 (I_p = 6), lag 012 (I_p = 7), lag 0123 (I_p = 8). I_p has a probability mass function $\prod_{j=1}^{8} \pi_j^{\delta_j(I_p)}$, where π_j denotes the inclusion probabilities of the predictor with a specific lag. so that $\sum_{j=1}^{8} \pi_j = 1$ and $\delta_j(.)$ is the Dirac function, $\delta_j(I_p) = \begin{cases} 1, if\ I_p = j \\ 0, if\ I_{p \neq j} \end{cases}$. A spike and slab prior distribution was assumed for the regression coefficients. The posterior probability of including each lag was computed, and the one with the highest probability was selected.

C- Wavelet spectrums analysis

Wavelet analysis was conducted to understand better the temporal dynamics of malaria transmission in association with climatic factors and to assess how their relationships vary over different time scales for each climatic zone [39]. We used the continuous wavelet transformation: $W_i(\tau, s) = \frac{1}{\sqrt{s}} \int_{-\infty}^{+\infty} f_i(t)\, ψ^* \left(\frac{t-\tau}{s}\right) dt$; $f_i\,(t)$ is the time series i at each specific month t where $i = 1$ indicates malaria incidence, $i = 2$ (rainfall) $and\ i = 3$ (LST); $ψ^*$ is the complex conjugate form of ψ, which is the Morlet wavelet function; τ is the translation parameter, determining the position of the wavelet along the time; and s is the scale parameter which approximates the time series period. Thus, the wavelet coefficients $W_i(\tau, s)$ decompose the time series i at different scales s along time locations τ. The wavelet power spectrum (WPS) was used to visualize the distribution of power and identify periodicity across different scales and positions in malaria incidence, rainfall, and LST. It was computed as $WPS_i(\tau, s) = |W_i(\tau, s)|^2$. The wavelet cross-spectrum quantifies the correlation between two non-stationary time series, *i* and *j*, i.e., of malaria with rainfall or LST. It is given by $W_{i,j}(\tau, s) = W_i^*\,(\tau, s) W_j(\tau, s)$. The cross coherence wavelet, $R_{i,j}(\tau, s)$ normalizes the wavelet cross-spectrum by the spectrum of each time series so that $R_{i,j}(\tau, s)$ is bounded by $0 \leq R_{i,j}(\tau, s) \leq 1$. It provides information about the frequency and temporal location that the time series are linearly correlated. The phase difference can obtain delays in the relationship between the two time series. $\varphi_{i,j}(\tau, s) = tan^{-1} \frac{Im(\langle W_{i,j}(\tau,s)\rangle)}{Re(\langle W_{i,j}(\tau,s)\rangle)}$ where $Im(\langle W_{i,j}(\tau, s)\rangle)$ and $Re(\langle W_{i,j}(\tau, s)\rangle)$ are the imaginery and real parts of the

smoothed cross-spectrum $W_{i,j}(\tau, s)$. It is expressed in radians, from −π to +π and displayed as arrow angles. An angle of 0 radians (an arrow pointing right) means the malaria time series and that of a climatic factor are in perfect synchrony (peaking and dipping simultaneously). An angle of π radians (an arrow pointing left) means the two-time series are perfectly out of synchrony (when one peaks, the other dips). Angles between 0 and π (or 0 and -π) show varying degrees of lead or lag between the two series. A significance level of 5% was for the statistical significance of the observed patterns. The wavelet analysis was carried out using the waveletComp package in R.
