# Supplementary material for: Impact of Climate Variability and Interventions on Malaria Incidence and Forecasting in Burkina Faso
Source: Int J Environ Res Public Health. 2024 Nov 8;21(11):1487. doi: 10.3390/ijerph21111487 (PMC11593955; doi:10.3390/ijerph21111487)
Supplement: Supplementary file 1 [file ijerph-21-01487-s001.zip › Supplementary material S2.pdf]

Table S1: Distribution of confirmed malaria cases by year during 2015-2021

Year	Malaria confirmed cases	Monthly cases per 1000			Percentiles 25th 50th 75th		
		Mean	Min	Max	25th	50th	75th
**2015**	23,852	9.91	1.76	32.71	4.68	7.50	13.96
**2016**	35,536	12.60	1.75	68.06	4.68	9.47	16.40
**2017**	35,104	11.69	2.00	29.25	7.25	10.37	15.91
**2018**	34,048	10.68	1.73	29.35	4.43	9.55	15.83
**2019**	21,954	6.75	0.01	23.98	2.18	5.96	9.94
**2020**	29,011	8.93	1.06	30.91	3.54	6.74	12.60
**2021**	29,806	8.85	1.73	29.68	4.37	6.87	11.86
**2015-2021**	29,902	9.92	0.01	68.06	4.43	7.89	13.84
