# Supplementary material for: Impact of Climate Variability and Interventions on Malaria Incidence and Forecasting in Burkina Faso
Source: Int J Environ Res Public Health. 2024 Nov 8;21(11):1487. doi: 10.3390/ijerph21111487 (PMC11593955; doi:10.3390/ijerph21111487)
Supplement: Supplementary file 1 [file ijerph-21-01487-s001.zip › Supplementary material S3.pdf]

Wavelet power and wavelet cross coherence by climatic zones

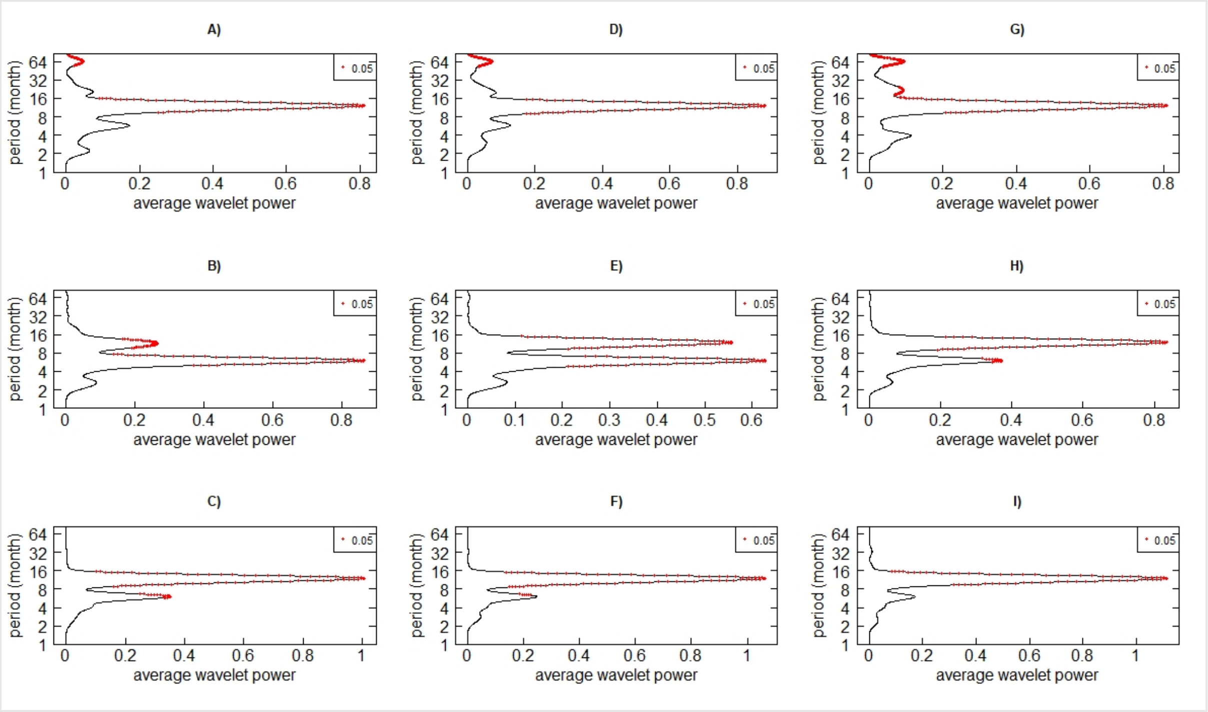

Figure S1: Average wavelet power of malaria incidence (A, D, G), LST (B, E, H), and rainfall (C, F, I), respectively, in the hot/arid, moderate, and cooler/wet zones.
